# Abnormal Ca^2+^ Signals in Reactive Astrocytes as a Common Cause of Brain Diseases

**DOI:** 10.3390/ijms23010149

**Published:** 2021-12-23

**Authors:** Schuichi Koizumi, Eiji Shigetomi, Fumikazu Sano, Kozo Saito, Sun Kwang Kim, Junichi Nabekura

**Affiliations:** 1 Department of Neuropharmacology, Interdisciplinary Graduate School of Medicine, University of Yamanashi, Yamanashi 409-3898, Japan; eshigetomi@yamanashi.ac.jp (E.S.); fsano@yamanashi.ac.jp (F.S.); ksaitoko@yamanashi.ac.jp (K.S.); 2 GLIA Center, Interdisciplinary Graduate School of Medicine, University of Yamanashi, Yamanashi 409-3898, Japan; 3 Department of Pediatrics, Faculty of Medicine, University of Yamanashi, Yamanashi 409-3898, Japan; 4 Department of Science in Korean Medicine, Graduate School, Kyung Hee University, Seoul 02447, Korea; skkim77@khu.ac.kr; 5 Division of Homeostatic Development, National Institute for Physiological Sciences, Okazaki 444-8585, Japan; nabekura@nips.ac.jp

**Keywords:** reactive astrocytes, Ca^2+^ signals, common pathology

## Abstract

In pathological brain conditions, glial cells become reactive and show a variety of responses. We examined Ca^2+^ signals in pathological brains and found that reactive astrocytes share abnormal Ca^2+^ signals, even in different types of diseases. In a neuropathic pain model, astrocytes in the primary sensory cortex became reactive and showed frequent Ca^2+^ signals, resulting in the production of synaptogenic molecules, which led to misconnections of tactile and pain networks in the sensory cortex, thus causing neuropathic pain. In an epileptogenic model, hippocampal astrocytes also became reactive and showed frequent Ca^2+^ signals. In an Alexander disease (AxD) model, *hGFAP*-R239H knock-in mice showed accumulation of Rosenthal fibers, a typical pathological marker of AxD, and excessively large Ca^2+^ signals. Because the abnormal astrocytic Ca^2+^ signals observed in the above three disease models are dependent on type II inositol 1,4,5-trisphosphate receptors (IP_3_RII), we reanalyzed these pathological events using IP_3_RII-deficient mice and found that all abnormal Ca^2+^ signals and pathologies were markedly reduced. These findings indicate that abnormal Ca^2+^ signaling is not only a consequence but may also be greatly involved in the cause of these diseases. Abnormal Ca^2+^ signals in reactive astrocytes may represent an underlying pathology common to multiple diseases.

## 1. Introduction

Under pathological conditions, astrocytes become reactive and exhibit a wide variety of responses, which are closely associated with the onset or development of several brain diseases. Thus, reactive astrocytes have received much attention, and researchers are continuing intense investigations to understand the nature and roles of these cells. The term reactive astrocyte includes a variety of astrocyte states. Researchers are attempting to identify simpler ways to describe these astrocytes, such as neurotoxic A1 astrocytes and neuroprotective A2 astrocytes [1]. However, reactive astrocytes are not so simple, and it turns out that the pattern of molecules expressed by reactive astrocytes varies greatly depending on the type of disease, degree of progression, and other factors [2]. In addition to changes in the expression of molecules, various indicators such as functional changes, anatomical changes, or a combination of the two must also be taken into account. One such indicator is aberrant Ca^2+^ signaling in astrocytes.

Astrocytes are non-excitable cells in terms of electrophysiological properties. However, with regard to Ca^2+^ signals, astrocytes are highly excitable and active. Astrocytes release so-called gliotransmitters such as ATP, glutamate, and D-serine [3,4], which are dependent on intracellular Ca^2+^ signals [5]. Astrocytes dynamically regulate neuronal functions via these gliotransmitters by controlling synaptic transmission [6]. The types and amounts of gliotransmitters vary in different pathological conditions, suggesting that various reactive astrocytes influence synaptic transmission in markedly different ways [7]. In addition to their rapid and acute control of synaptic activities, astrocytes also play an important role in the control of long-term neuronal activities. Reactive astrocytes produce a wide variety of synaptogenic molecules such as thrombospondins [8], hevin [9], and glypicans [10], for which astrocytic Ca^2+^ has also an important role. These molecules lead to highly uncontrolled synapses and affect neuronal activities and network connectivity. Astrocytes also affect long-term neuronal activities by forming synapses [11,12]. In early developmental stages [13], pathological conditions in adults [14], and the process of learning [15], astrocytes eliminate synapses by phagocytosis, thereby producing long-lasting network activities. Therefore, phenotypical changes in astrocytes in various pathological conditions are closely associated with both acute and chronic neuronal activities. Interestingly, Ca^2+^ signals are greatly enhanced in these reactive astrocytes [16]. This suggests that aberrant Ca^2+^ signals in reactive astrocytes may be a common characteristic of astrocyte-mediated brain diseases. In this review, we will focus on aberrant Ca^2+^ signals as a phenotype of reactive astrocytes. We show some examples of brain dysfunctions such as neuropathic pains [11,12,17,18], epileptogenesis [19,20,21], and Alexander disease (AxD) [22], where aberrant Ca^2+^ signals in astrocytes are commonly observed and are highly involved in the cause of these pathogenesis.

## 2. Neuropathic Pain and Excess Ca^2+^ Signals in Astrocytes of the Primary Sensory (S1) Cortex

**Figure 1:**
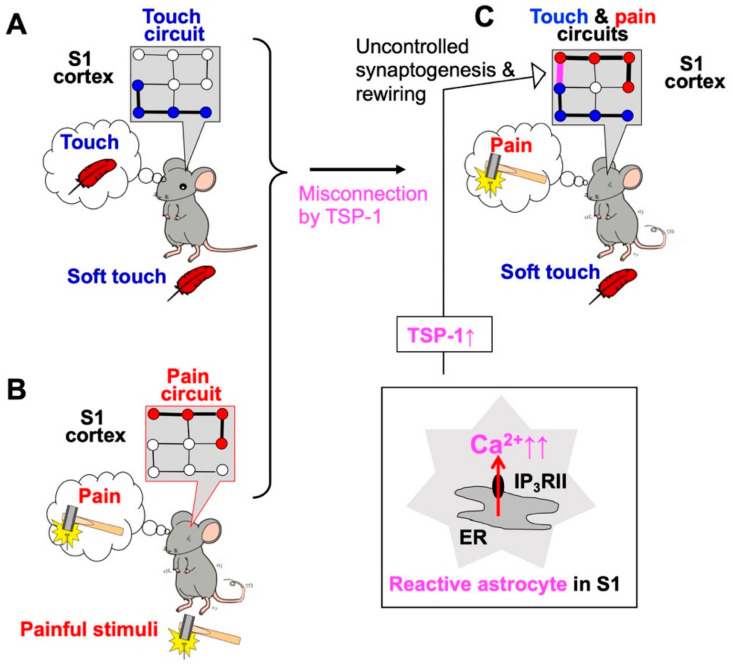
Abnormal Ca^2+^ signals in primary somatosensory (S1) cortex astrocytes cause neuropathic pain and allodynia. Soft touch stimulation (**A**) and pain stimulation (**B**) activate touch circuits and pain circuits, respectively. In a model of neuropathic pain induced by sciatic nerve ligation, S1 astrocytes become reactive and exhibit excess Ca^2+^ signaling via type II inositol 1,4,5-trisphosphate receptors (IP_3_RII) (**C**). Reactive astrocytes in the S1 cortex produce synaptogenic molecules such as thrombospondin-1 (TSP-1) and cause excessive, uncontrolled synaptogenesis and network rewiring of S1 circuits (**C**, arrow), thereby leading to mechanical allodynia.

Neuropathic pain is an intractable chronic pain condition, and one of its main symptoms is mechanical allodynia. The causes of neuropathic pain have remained unknown for many years, but advances in glial research are beginning to shed light on some of its underlying issues. One study that has made a substantial contribution is that of Tsuda and colleagues, who found that abnormalities in spinal microglia expressing P2X4 receptors led to abnormal synaptic transmission, thereby resulting in mechanical allodynia [23]. Since then, research on spinal microglia and neuropathic pain has progressed considerably, and many important discoveries have been made. However, many patients still suffer from neuropathic pain, suggesting the need for other approaches to determine the molecular pathogenesis of neuropathic pain. In humans, it is reported that neuropathic pain is associated with infra-slow oscillatory activities in some brain regions such as somatosensory thalamus and primary somatosensory cortex, which interestingly, occur at frequencies similar to Ca^2+^ waves in reactive astrocytes [18]. This finding strongly suggests that brain astrocytes would be involved in the pathogenesis of neuropathic pain. Our research has focused on abnormalities in the neural network of the primary somatosensory cortex (S1) and has shown that during the development of mechanical allodynia, synaptic remodeling in the S1 cortex is enhanced. This leads to the reorganization of the S1 network and misconnection of the pain and touch circuits. Once these circuits are reorganized, they are maintained, resulting in chronic neuropathic pain [11,12]. Reactive astrocytes and their synaptogenic molecule thrombospondin-1 (TSP-1) play essential roles in this type of synaptic and network remodeling (Figure 1). Importantly, reactive astrocytes are highly associated with aberrant Ca^2+^ signals, which are dependent on Ca^2+^ release from type II inositol 1,4,5-trisphosphate receptors (IP_3_RII). To clarify whether such astrocytic Ca^2+^ signaling is a result or a cause of synaptic remodeling and neuropathic pain, we examined IP_3_RII-deficient (IP_3_RIIKO) mice and found that all of the above events, i.e., abnormal Ca^2+^ signals, TSP-1 production, synapse remodeling, and mechanical allodynia, were abolished in these mice. Thus, it is concluded that abnormal Ca^2+^ signals in reactive astrocytes may cause a series of pain signaling cascades leading to mechanical allodynia [11,12].

## 3. Other Brain Diseases and Aberrant Astrocytic Ca^2+^-mediated Synapse Remodeling

Similar astrocyte-mediated Ca^2+^ signals and synapse remodeling were also observed in the other brain regions, which are involved in different brain disorders. For example, activation of striatal medium spiny neurons (MSNs) results in GABA release, which stimulates GABA_B_ receptors and Ca^2+^ elevation in adjacent astrocytes. Then, striatal astrocytes produce the synaptogenic molecule TSP-1 and boost excitatory synapse formation via an a2d-1-mediated mechanism. This in turn increases the firing of MSNs, which results in hyperactivity with disrupted attention phenotypes in mice. When the GABA_B_ receptor-mediated Ca^2+^ responses were mimicked by stimulating Gi-designer receptors exclusively activated by designer drugs (Gi-DREADD) gated by clozapine-N-oxide (CNO), all subsequent responses, i.e., TSP-1 production, excessive synaptogenesis, and abnormal behaviors, were reproduced [24]. Therefore, aberrant Ca^2+^ signals in striatal astrocytes could also be a cause of the abnormal behavior, i.e., hyperactivity and disturbances of attention.

In addition, cocaine-induced abnormal behaviors such as drug seeking and relapse after withdrawal are mediated by aberrant Ca^2+^ signals in astrocytes in the nucleus accumbens shell (NAcSh). The administration of cocaine increases aberrant Ca^2+^ signals in NAcSh astrocytes, which stimulates the production of the synaptogenic molecule TSP-2, leading to the activation of its receptor a2d-1 and the generation of AMPA receptor-silent glutamatergic synapses [25]. Importantly, the cocaine-evoked formation of glutamatergic silent synapses in NacSh functions as a memory trace of cocaine experience, and thus, the aberrant Ca^2+^ signals in astrocytes could be a cause also of cue-associated memory traces that promote cocaine relapse.

In the examples shown in Section 2 and Section 3, aberrant Ca^2+^ signals in astrocytes produce an excess of synaptogenic molecules such as TSP-1, leading to uncontrolled synapse remodeling and network remodeling. Therefore, it is suggested that aberrant astrocytic Ca^2+^ signals are decoded in the form of the production of a synaptogenic molecule and cause various different brain disorders. With regard to astrocytic synaptogenic molecules, many other molecules besides TSPs have been reported. Since Barres’s group first showed that astrocytes dramatically control synapse formation, maturation, and even elimination, as well as synaptic efficacy by releasing soluble proteins [26,27], a range of astrocyte-secreted molecules that can induce synapse remodeling during development has been identified: brain-derived neurotrophic factor (BDNF), cholesterol, glypicans, hevin, SPARC, transforming growth factor β (TGF-β), tumor necrosis factor α (TNF-α), TSPs [28,29] .(Briefly, BDNF released from astrocyte-like non-neuronal cells, called supporting cells, in the vestibular organ promotes synapse formation between hair cells and primary vestibular sensory neurons [30]. Cholesterol is the first identified astrocyte-derived molecule that induces excitatory synapse formation in retinal ganglion cell (RGC) cultures [31]. Glypicans 4 and 6 promote the formation of postsynaptically functional excitatory synapses by increasing the levels of AMPA receptors on the surface of the postsynaptic membrane [10]. Hevin induces the formation and/or maturation of retinocollicular and thalamocortical synapses, whereas SPARC antagonizes the synaptogenic effect of hevin [32,33]. Chordin-like 1 drives synapse maturation by increasing GluA2 AMPA receptors [34]. TGF-β also showed synaptogenic properties in cultured cortical neurons and in neuromuscular junction [35,36], and TNF-α increases the surface expression of AMPA receptors to maintain synaptic strength and contributes to the homeostatic activity-dependent refinement of neural circuits during development [37,38]. Finally, TSPs are suggested to have a primary role in astrocyte-mediated excitatory synapse formation [28,39]. Astrocytes express high levels of TSP-1 and -2 during development when neural circuits are massively forming, and double knockout mice have fewer excitatory synapses in the brain as compared to wild-type mice [8]. Adhesion molecules also have important roles in the formation of excitatory synapses by stimulating protein kinase C-mediated signals [40]. Astrocytes express many types of molecules that induce the formation and maturation of synapses. The identification of the mechanisms by which astrocytes use these molecules differentially will require further research [41].

## 4. Epileptogenesis and Aberrant Ca^2+^ Signals in Epileptogenic Astrocytes

**Figure 2:**
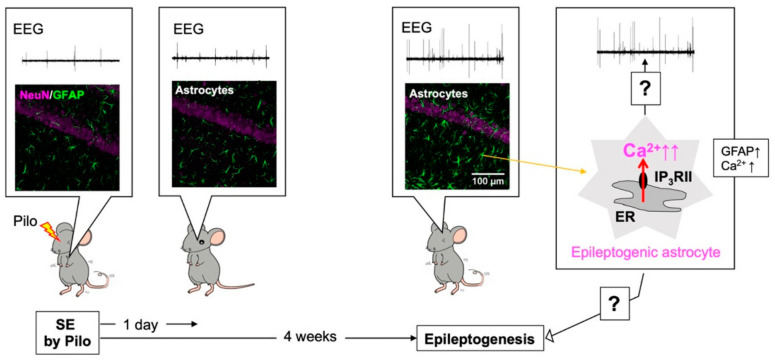
Abnormal Ca^2+^ signals in hippocampal astrocytes cause epileptogenesis. Four weeks, but not 1 day, after pilocarpine (Pilo)-evoked status epilepticus (SE), hippocampal astrocytes became reactive and showed increased glial fibrillary acidic protein (GFAP) expression and frequent abnormal Ca^2+^ signaling (right cartoon). Reactive astrocytes were highly associated with the development of epileptogenesis as assessed by ictal spikes (electroencephalogram; EEG) or seizure susceptibility to Pilo and, thus, they were named “epileptogenic astrocytes”. Epileptogenesis appeared dependent on abnormal Ca^2+^ signaling in epileptogenic astrocytes. The mechanism by which epileptogenic astrocytes cause epileptogenesis is a subject for future research.

Astrocytes are known to be reactive in the brain of epilepsy patients [42] and animal models of epilepsy [43], and a link between the molecular pathogenesis of epilepsy and reactive astrocytes has long been suggested. Epileptic seizures and ictogenesis result from abnormal and excessive excitability of neurons and networks and astrocytic dysregulation of neuronal excitability, including reduced uptake of extracellular glutamate by GLT-1 [44] and K^+^ by Kir4.1 [45]. Numerous reports have examined the association between epileptic seizures and dysfunction of these astrocytic molecules, including Ca^2+^ [46]. Epilepsy not only results from overexcitation of neurons but also is a condition in which excitation occurs repeatedly. Thus, in addition to the clarification of ictogenesis and neuronal overexcitation, the mechanism by which epileptic seizures are repeated needs to be elucidated, or in other words, the mechanism that makes epilepsy more likely to occur needs to be determined [19,20]. This situation, in which the brain becomes prone to recurrent epileptic seizures is called epileptogenesis. We examined epileptogenesis and reactive astrocytes and found that excess Ca^2+^ signals in reactive astrocytes cause epileptogenesis [21]. Pilocarpine (Pilo) was used to induce epileptogenesis. When Pilo is injected in mice, the animals soon show a strong epileptic seizure, known as status epilepticus (SE). After SE, the mice become progressively more sensitive to Pilo and more prone to epileptic seizure, and 4 weeks after SE, they become completely epileptogenic and show spontaneous ictal spikes (Figure 2). We analyzed the time course of glial activation in the hippocampus and found that after SE, microglia were immediately activated, but this was transient, and the microglia quickly returned to their original state. However, astrocytes gradually became reactive, which is highly consistent with the time course of epileptogenic acquisition. Thus, we hypothesized that reactive astrocytes are involved in epileptogenesis and tentatively named them “epileptogenic astrocytes”. We screened characteristic features of epileptogenic astrocytes and found that they showed very frequent, intense, and wide-spreading Ca^2+^ signals. These abnormal Ca^2+^ signals were mediated by IP_3_RII. We thus determined whether Pilo-evoked epileptogenic astrocytes, excess Ca^2+^ signals, and epileptogenesis were observed in IP_3_RIIKO mice and found that all epileptic events were abolished in these mice. Therefore, we concluded that the abnormal Ca^2+^ signals observed in reactive astrocytes cause epileptogenesis [21].

## 5. Alexander Disease (AxD) and Aberrant Ca^2+^ Signals in AxD Astrocytes

**Figure 3:**
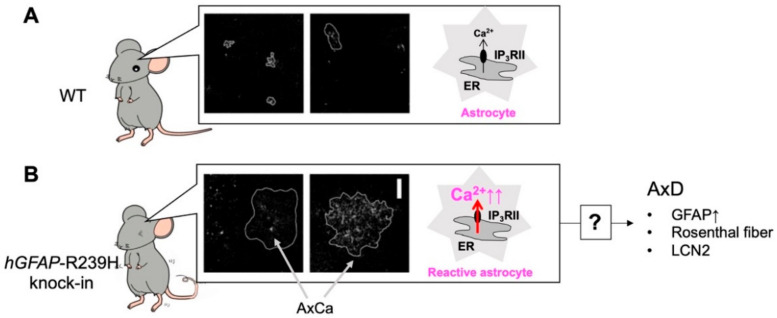
Abnormal Ca^2+^ (AxCa) signals in reactive astrocytes cause Alexander disease (AxD). Unlike in WT mice (**A**), in *hGFAP*-R239H knock-in mice, hippocampal astrocytes are reactive and show abnormal extremely large Ca^2+^ signals called “AxCa” signals (**B**). AxCa signals in AxD astrocytes are mediated by type II inositol 1,4,5-trisphosphate receptors (IP_3_RII) and are abolished in AxD-IP_3_RII knockout (KO) mice. Importantly, typical indicators of AxD such as an increase in glial fibrillary acidic protein (GFAP), the formation of Rosenthal fibers, and the induction of lipocalin 2 (LCN2) are dramatically decreased in AxD-IP_3_RIIKO mice. Although the mechanisms underlying AxCa signal-mediated AxD pathology remain largely unknown, AxCa signals in AxD astrocytes are a cause of AxD.

AxD is a very rare neurodegenerative disease that was first described by Steward Alexander in 1949 [47]. AxD is caused by mutation of the gene encoding glial fibrillary acidic protein (GFAP), an astrocyte-specific intermediate filament. In AxD patients and animal models, astrocytes become reactive and show intracellular aggregates called Rosenthal fibers including GFAP, ab-crystallin, vimentin, and heat shock protein27, which is a typical pathological feature [48]. Since AxD is a primary astrocytic disease, it is speculated that astrocytic dysfunction triggers it, which in turn causes severe neurological damage. Despite a great deal of vigorous research, little is known about how Rosenthal fibers-containing astrocytes become abnormal and the mechanisms involved in AxD development. In addition, the symptoms of AxD have multiple clinical forms, and its onset also varies from newborns to adults. It also remains unclear how mutations in *GFAP* cause such diverse and severe neurodegenerative diseases. We conducted a detailed analysis of AxD astrocytes using a mouse model of AxD overexpressing a mutated human *GFAP* gene (*hGFAP*-R239H knock-in mice) [49]. AxD astrocytes are reactive, and interestingly, also show abnormal Ca^2+^ signals, called AxCa (aberrant extra-large Ca^2+^) signals (Figure 3) [22]. AxCa signals are highly associated with AxD pathology and are caused by the activation of IP_3_RII. It was not known whether AxCa signals are a cause or a result of AxD. To determine this, we crossed the AxD mice with IP_3_RIIKO mice (AxD–IP_3_RIIKO). AxD-IP_3_RIIKO mice dramatically decreased AxCa signals as well as aggregated GFAP accumulation, a typical pathological indicator of AxD. Therefore, we concluded that abnormal Ca^2+^ signals in reactive astrocytes (AxD astrocytes) are a cause of AxD pathology [22].

Although aberrant Ca^2+^ signals mediated by IP_3_RII in AxD astrocytes were found to be associated with the molecular pathogenesis of AxD, it remains unclear whether these are cell-autonomous actions of astrocytes or whether they involve the communication with other cells, such as neurons, microglia, and blood vessels. Recently, in iPS cell-derived astrocytes from AxD human patients, aberrant Ca^2+^ signals were also observed [50]. AxD iPS cell-derived astrocytes showed decreased Ca^2+^ propagation, which was due to impaired extracellular release of ATP. In this experimental system, only iPS cell-derived astrocytes are present, and there is no communication with other types of cells. Therefore, aberrant Ca^2+^ signaling in AxD astrocytes occurs as a cell-autonomous action of astrocytes, suggesting that *GFAP* mutations would be translated into abnormal Ca^2+^ signals in astrocytes, leading to the development of AxD. However, the molecular mechanisms and cascades leading from aberrant Ca^2+^ signals to AxD remain unclear. By focusing on Ca^2+^ abnormalities in astrocytes, future research on AxD is expected to make significant progress.

## 6. Complexity of Ca^2+^ Signals in Astrocytes

In this manuscript, we have simply described astrocyte dysfunctions in terms of “aberrant astrocyte Ca^2+^ signals”. However, Ca^2+^ signal shows more complex spatiotemporal variations than we thought, and the mechanisms by which these occur are different. For example, Ca^2+^ signals have three different patterns in striatal astrocytes, defined as global Ca^2+^ signals, local Ca^2+^ signals, and Ca^2+^ microdomains [51]. Global Ca^2+^ signals are well observed in the somata of astrocytes and are mainly due to Ca^2+^ release from IP_3_RII, followed by Ca^2+^ entry via store-operated Ca^2+^ entry mechanisms. In addition, Ca^2+^ shut-off mechanisms mediated by the endoplasmic reticulum Ca^2+^ pump, the plasma membrane Ca^2+^ pump, and the Na^+^/Ca^2+^ exchanger also affect Ca^2+^ signals there. On the contrary, Ca^2+^ microdomains are mainly observed in the fine processes of astrocytes and are rather produced by Ca^2+^ entry via Ca^2+^ entry channels such as TRPA1 [52]. Of course, these classifications are not exhaustive, but it should be noted that even healthy astrocytes in the same brain region show such functional heterogeneity in Ca^2+^ signals. In addition, the word “aberrant Ca^2+^ signal” means not only an abnormal increase but also an abnormal decrease in Ca^2+^ signals. However, other than the fact that differences in the spatiotemporal pattern of Ca^2+^ signals in astrocytes have a significant effect on astrocyte function, research in this field has not progressed, and the actual situation is not well understood. Therefore, in future studies, we should investigate and categorize the aberrant Ca^2+^ signals in astrocytes from the point of view of temporal and spatial differences as well as of qualitative differences, so to classify them more precisely.

## 7. Conclusions and Future Perspectives

**Figure 4:**
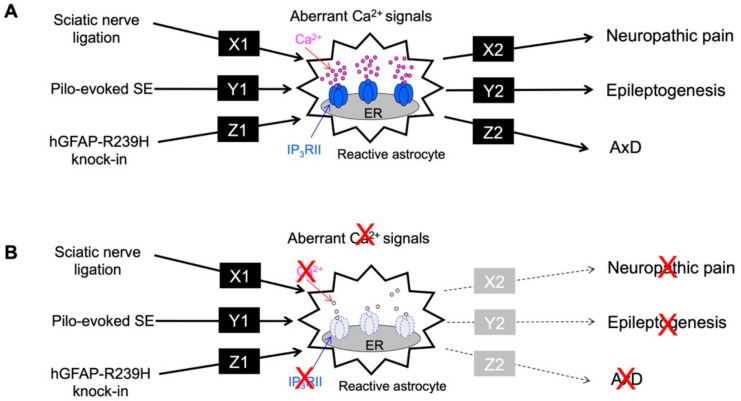
Summary of aberrant Ca^2+^ signals and neurodegenerative disorders. (**A**). Sciatic nerve ligation (neuropathic pain model), Pilo-evoked SE (epileptogenesis model), and hGFAP-R239H knock-in (AxD model) caused a common phenomenon of aberrant Ca^2+^ signals in reactive astrocytes, although each pathway (X1, Y1 and Z1) is completely different. In addition, the aberrant astrocytic Ca^2+^ signal observed in each pathological model causes neuropathic pain, epileptogenesis, and AxD via different pathways (X2, Y2 and Z2). Molecules or signals of X1-Z1, X2-Z2 are unknown. (**B**). The aberrant astrocytic Ca^2+^ signal observed in each pathological model was mediated by IP_3_RII. Therefore, when IP_3_RIIKO mice were used, the aberrant Ca^2+^ signal induced by sciatic nerve ligation, SE, and hGFAP-R239H knock-in, respectively, was abolished. Importantly, the symptoms of neuropathic pain, epileptogenesis, and AxD also disappeared. Therefore, aberrant Ca^2+^ signals seen in astrocytes may be one of the common causes of these brain disorders.

As shown in the above examples, astrocytes show common characteristics in three very different diseases, i.e., they appeared reactive and exhibited abnormal Ca^2+^ signals, and very importantly, aberrant Ca^2+^ signaling was found to be a cause of these diseases. As shown in Figure 4A, the mechanisms by which abnormal Ca^2+^ signals are induced in various diseases differ (Figure 4A, X1, Y1, and Z1), and the mechanisms by which abnormal Ca^2+^ signals affect the development and progression of various diseases also markedly differ (Figure 4A, X2, Y2, and Z2). However, evidence strongly suggests that each disease may develop and progress via abnormal astrocytic Ca^2+^ signaling or that abnormal astrocytic Ca^2+^ signals may be a common underlying dysfunction in neurodegenerative diseases, because inhibition of these abnormal Ca^2+^ signals by deletion of IP_3_RII dramatically reduced all three disease states (Figure 4B). Thus, the elucidation of the mechanisms of astrocytic Ca^2+^ signaling, especially abnormal Ca^2+^ signaling, is a fascinating subject that may reveal common principles of neurodegenerative diseases and directly lead to the development of high-impact therapeutic strategies common to many brain diseases. In the future, it will be necessary to clarify the actual Ca^2+^ abnormalities at the molecular level by comprehensive analyses, such as single-cell RNA sequencing and proteome analysis, and to analyze the mechanisms responsible for Ca^2+^ signaling abnormalities (Figure 4, X1, Y1, and Z1) and the diseases resulting from Ca^2+^ signaling abnormalities (Figure 4, X2, Y2, and Z2). In addition, although this review is focused on abnormal Ca^2+^ signaling in astrocytes, the nature of the “abnormality” in each disease is different. The degree to which Ca^2+^ signals are abnormal in astrocytes also needs to be carefully studied.

## Data Availability

The data presented in this study are available on request from the corresponding author.
